# Epidemiological situation of schistosomiasis in 16 districts of Burkina Faso after two decades of mass treatment

**DOI:** 10.1371/journal.pntd.0012858

**Published:** 2025-02-06

**Authors:** Dramane Zongo, Josiane Marie Agathe Tiendrebeogo, Wendegoudi Mathias Ouedraogo, Mohamed Bagayan, Sidwaya Hamed Ouedraogo, Clarisse Bougouma, Christophe Nassa, Mamadou Serme, Dieudonné Naré, Adama Zida, Ibrahim Sangaré, Micheline O. Ouedraogo, Patricia Houck, Steven Reid, Anna E. Phillips, Jennifer Magalong, Angela M. Weaver, Yaobi Zhang, Joseph Kouesyandé Soubeiga

**Affiliations:** 1 Research Institute for Health Sciences, Ouagadougou, Burkina Faso; 2 Programme national de lutte contre les maladies tropicales négligées, Ministère de la santé, Ouagadougou, Burkina Faso; 3 University Joseph Ki-Zerbo of Ouagadougou, Ouagadougou, Burkina Faso; 4 Direction de la protection de la santé de la population, Ministère de la santé, Ouagadougou, Burkina Faso; 5 Helen Keller International, Ouagadougou, Burkina Faso; 6 Centre National de Recherche et de Formation sur le Paludisme, Ministère de la santé, Ouagadougou, Burkina Faso; 7 Laboratoire des pathogènes émergents et réémergent (LaPathER), Université Nazi BONI, Bobo-Dioulasso, Burkina Faso; 8 Département des Laboratoires, Centre Hospitalier Université Souro SANOU, Bobo-Dioulasso, Burkina Faso; 9 Laboratoires de Recherche, Équipe Parasitologie - Mycologie -Entomologie, Centre Muraz, Bobo Dioulasso, Burkina Faso; 10 Helen Keller International, New York, New York, United States of America; 11 FHI 360, Washington DC, United States of America; 12 Direction générale de la santé et l’hygiène publique, Ministère de la santé, Ouagadougou, Burkina Faso; University of Florida, UNITED STATES OF AMERICA

## Abstract

**Background:**

After two decades of mass drug administration (MDA) for schistosomiasis in Burkina Faso, an impact assessment was conducted in 16 health districts (HDs) between February 2023 to March 2024 to determine the epidemiological situation of schistosomiasis in school-aged children and facilitate the decision for sub-district level treatment decision.

**Methodology/Principal findings:**

A cross-sectional cluster survey was conducted with randomly selected children aged 5–14 years. Haemastix reagent strips were used to detect microhaematuria in urine, and urine filtration was used to detect and quantify *Schistosoma haematobium* eggs in children with microhaematuria. The Kato-Katz was used to detect and quantify *S. mansoni* eggs in fecal samples. The prevalence of infection and the prevalence of heavy-intensity (HI) infections were calculated. The overall prevalence of schistosomiasis was 2.4% (range: 0–11.1%) in 16 HDs, with the overall prevalence of HI infection of 0.9% (range: 0–4.2%). Four HDs (Tenkodogo, Batié, Sig-Nonghin, and Karangasso-Vigué) had a prevalence of HI infection from 1.3% to 4.2%. Forty-seven health areas still had an overall prevalence of 1.4–62.5%, with seven health areas in five HDs >10%, and 29 health areas had a prevalence of HI infection from 1.4% to 22.9%. Boys had a higher prevalence of HI infections than girls. The prevalence of microhaematuria at 5.8% was significantly higher than that of *S. haematobium* infection by urine filtration at 2.3%, and Haemastix results were significantly associated with the intensity of infection.

**Conclusions/Significance:**

The results showed that progress has been made in eliminating schistosomiasis as a public health problem in the 16 HDs in Burkina Faso after two decades of mass treatment. However, hotspots of infections remain, with 29 health areas having a prevalence of HI infection above the 1% threshold. The results provide evidence for planning targeted sub-district-level treatment.

## Author summary

Burkina Faso is endemic for schistosomiasis in all health districts and has been conducting mass treatment with praziquantel at the district level in the last two decades, targeting school-aged children regularly and at-risk adults where prevalence was high. We conducted an impact assessment in 16 health districts in 2023–2024 to assess the current epidemiological situation and provide evidence to adjust treatment strategies at the sub-district level. Children aged 5–14 years were selected randomly from 320 schools. Urine samples were tested for urogenital schistosomiasis using urine filtration and for microhaematuria using Haemastix urinalysis reagent strips. Stool samples were tested for intestinal schistosomiasis using Kato-Katz. The overall prevalence of schistosomiasis was 2.4% in 16 health districts, with the overall prevalence of infection with heavy intensity at 0.9%. The prevalence of microhaematuria was 5.8%. The results showed good progress in eliminating schistosomiasis as a public health problem in the 16 health districts after two decades of mass treatment. There were, however, some hotspots of infection, as seven health areas in five health districts had an overall prevalence of >10%, and a total of 29 health areas had a prevalence of infection with heavy intensity above the 1% threshold for elimination as a public health problem. The results provide evidence for planning targeted sub-district-level treatment.

## Introduction

Schistosomiasis is one of the world’s major transmissible diseases with significant implications for public health and socio-economic development in developing countries [1]. It is a chronic waterborne parasitosis caused by a hematophagous worm (trematode) called schistosome or bilharzia. The disease is transmitted by intermediate hosts (freshwater molluscs), which are aquatic, hermaphroditic, and oviparous gastropod molluscs [2,3]. There are two major forms of schistosomiasis in sub-Saharan Africa: urogenital schistosomiasis caused by *Schistosoma haematobium* and intestinal schistosomiasis caused by *S. mansoni*. Urogenital schistosomiasis is the most common form, representing about two-thirds of cases [4]. As a neglected tropical disease, the World Health Organization (WHO) estimated that more than 250 million people worldwide are infected by schistosomiasis, with approximately 90% in Africa [3]. The current burden of schistosomiasis remains high and could exceed 1.6 to 4.2 million disability-adjusted life years [1,5,6]. The disease is endemic in communities related to agricultural development and increased irrigation networks. Lack of hygiene and play areas, especially in overflowing water, make children particularly vulnerable to schistosomiasis. The schistosomiasis control strategy focuses on periodic large-scale population treatment with praziquantel [2,7].

In Burkina Faso, *S. haematobium* is predominantly found in all regions of the country, and *S. mansoni* is present in the southern and western parts of the country [8,9]. The national schistosomiasis and soil-transmitted helminths control program started in 2004, administering praziquantel biennially to school-aged children (SAC) at the district level [10,11]. After a decade of mass drug administration (MDA), great progress was made in reducing the prevalence among SAC [11]. However, this disease remained a public health problem [12,13]. Reports indicated that more than one million children were still infected with persistent hotspots in the Sahel region for urogenital schistosomiasis (around 30% prevalence in 2016) and in the southern and western regions for the intestinal form (around 20% in 2016) [11]. Following an expert review in 2013, the national treatment strategy was aligned with the WHO recommendations based on prevalence data and the local context [2,14]. Treatment was implemented in 2015 twice a year in Centre Est, once a year in the regions of Boucle du Mouhoun and Sahel, and every other year in all other regions. In 2019, a second expert review recommended that Burkina Faso conduct assessments to review the impact of multiple rounds of MDA and fill the data gaps to transition to sub-district level treatment.

During February 2023 and March 2024, a re-assessment was conducted in 16 health districts (HDs). This study aimed to determine the prevalence and intensity of infection of schistosomiasis among SAC after two decades of mass treatment. The results will enable the national program to refine treatment strategies at the sub-district (health area) level in these HDs, and to improve educational approaches, which are very important in Burkina Faso’s specific social contexts.

## Materials and methods

### Ethics statement

The National Program team on Neglected Tropical Diseases was authorized by the Ethics Committee of Health Research, Ministry of Health of Burkina Faso, to conduct this survey (Reference number 2022-11-231). School authorities, teachers, parents/guardians, and participants were informed about the objectives, procedures, and potential risks and benefits of the study. Written informed consent was obtained from children’s parents or legal guardians. Oral assent was obtained from children.

### Study settings

Burkina Faso is a landlocked Sahelian country with a surface area of around 274,200 km^2^, bordered to the north-west by Mali, to the north-east by Niger, to the south-east by Benin and to the south by Côte d’Ivoire, Ghana and Togo. The population of Burkina Faso is estimated at 22,916,561 in 2023, representing a density of around 73.4 inhabitants/ km^2^, and about 77.3% of the population lives in rural areas with agriculture as their main activity. Burkina Faso’s population is characterized by its extreme youth, with 45.96% of the population under 15 years [15]. The country has a tropical Sudanian climate, with average annual rainfall ranging from 300 mm in the north to over 1,200 mm in the south-west. The country is exposed to permanent risks of flooding, harmattan drought, and sometimes very high temperatures. The vegetation is of the Sudano-Sahelian type. The hydrographic network comprises numerous rivers, the main ones being the Mouhoun, Nakambé, Nazinon, and Comoe rivers, as well as numerous dams and hydro-agricultural facilities [16].

Burkina Faso’s health system is subdivided into 13 Regional Directorates, corresponding to the 13 administrative regions, 70 HDs, and 2,222 community health facilities representing health areas. Sixteen HDs in Burkina Faso were selected for the assessment based on the absence of recent epidemiological data (2013–2021) and the feasibility of the field survey in the context of insecurity. In addition, the HDs selected have regularly conducted at least five rounds of MDA with effective coverage (≥75%). These 16 HDs are in the regions of Boucle du Mouhoun (1), Centre (2), Center Est (3), Centre Oust (2), Hauts Bassins (3), Plateau Central (3), and Sud Ouest (2) respectively. The study was carried out across 16 HDs 1–4 years after the last MDA, where a total of 8–13 rounds of MDA had been conducted for SAC in each HD, with irregular MDA for adults in most districts (0–8 rounds). S1 Table summarises the MDA rounds and coverage in the 16 HDs.

### Selection of survey sites in health districts

A cross-sectional survey was conducted in the 16 HDs between February 2023 and March 2024. A two-stage cluster sampling was used, surveying 20 schools per HD. First, ten health areas (sub-districts) were selected systematically from each HD according to probability proportional to the health area population size. Then, two schools were selected randomly from each selected health area. This resulted in 320 schools in 160 health areas across the 16 HDs.

### Selection of school children in schools

School teachers, children, community leaders, and parents were explained the purpose of the survey, the survey protocol and sample collection process, and the requirement for informed consent. Only children aged 5–14 years with parental consent were selected and included in the sampling frame. In each school, 24 children (12 boys and 12 girls) aged 5–14 years were selected randomly and identified by unique codes. In practice, two boys and two girls were randomly selected from each of the six grades. Where schools had fewer than six grades, additional children were allocated to existing grades to make it up to 24 children. Each selected child received two containers to collect one urine and one stool sample. The urine and stool samples were collected from 8 to 10 am. The samples were brought to the laboratory in a cool box.

### Sample examination

Haemastix reagent strips (Siemens) were used to detect microhaematuria as a proxy for *S. haematobium* infection for all urine samples [17]. Haemastix results were considered positive for microhematuria if the test showed ‘trace hemolyzed’, ‘+’, ‘++’, or ‘+++’. For urine samples with microhaematuria, urine filtration (UF) was conducted to quantify *S. haematobium* eggs in the urine [18]. The urine container was vigorously shaken, and 10 milliliters of urine were taken and filtered through a polycarbonate membrane filter (pore size 12 µm, using a urine filtration kit (Sterlitech Corporation, Kent, USA) and examined microscopically for the presence of *S. haematobium* eggs by specialized medical laboratory technicians. Stool samples were analyzed by the Kato-Katz method using the Kato-Katz kits (Sterlitech Corporation, Kent, USA) [19]. The Kato-Katz method has low sensitivity for low-intensity infections, which may underestimate the prevalence [20,21]. However, it is still the “gold standard” diagnostic tool recommended for evaluating schistosomiasis programs [22]. In our surveys, two thick smear slides from each stool sample were used and examined microscopically by experienced laboratory technicians to identify and quantify eggs of *S. mansoni*.

### Data collection and analysis

An electronic data collection tool, ESPEN Collect, was used to collect socio- demographic information and laboratory results of each child. The data were downloaded, cleaned, and analyzed using the IBM SPSS Statistics (version 23). We also extracted the prevalence data from survey sites in any of the 16 HDs that had been sampled in previous surveys and grouped them into three time periods: 2004–2005 (baseline mapping), 2008–2013 (first impact assessment), and 2016–2019 (second impact assessment), for comparison with the current survey data.

Egg counts for *S. haematobium* for each child were calculated as the number of eggs per 10 ml of urine (eggs/10ml), assuming samples that were Haemastix-negative and not tested by UF were egg-negative. Egg counts for *S. mansoni* were calculated for each child as the number of eggs per gram of faeces (epg) by multiplying the average egg counts from two slides by 24. The intensity of infection for an individual child was classified as heavy (≥50 eggs/10 ml) or low (<50 eggs/10 ml) for *S. haematobium* infection and heavy (≥400 epg), moderate (100–399 epg) and low (<100 epg) for *S. mansoni* infection. The unadjusted prevalence of infection, the prevalence of heavy intensity (HI) infection, and the arithmetic mean of egg counts among all children examined, with 95% confidence intervals (CI) from all SAC examined, were calculated by sex, age, health areas, and HDs.

The chi-squared test was used to compare the prevalence between tests and groups as indicated. The chi-squared test for trend was performed using the online testing tool available at Epitools to explore trend in prevalence between years of the surveys [23]. The correlation between Haemastix results and egg counts for *S. haematobium* was tested by Pearson correlation. GPS coordinates collected for each school were used to plot the locations and point prevalence using the ArcGIS version 10.8.2 (ESRI, Redlands, California, United States). Spatial analysis was conducted to predict prevalence distribution across the surveyed districts using ordinary kriging with a circular semivariogram model in the Geostatistical Analyst, and potential hotspots were identified using the hotspot analysis (Getis-Ord Gi*) tool in the ArcGIS.

## Results

### Prevalence

In total, 7,664 urine samples and 7,669 stool samples in 320 schools in 160 health areas across 16 HDs were examined for *S. haematobium* and *S. mansoni* infections (Table 1). Overall, the prevalence of *S haematobium* infection by UF was 2.3% (2.0–2.7%), and the prevalence of microhaematuria was 5.8% (5.3–6.3%). *S. mansoni* infection was found in only three districts (Batie, Do, and Lena), with prevalences of 0.2%, 0.6%, and 0.2%, respectively. The overall prevalence of infection with any species was 2.4% (95% CI: 2.1–2.8%).

**Table 1:** Results of re-assessment of schistosomiasis prevalence in 16 districts in 2023-2024 in Burkina Faso.

Districts surveyed	Nunber of children tested for *S. haematobium*	Number of children positive with *S. haematobium* by UF	Prevalence (%) of *S. haematobium infection* by UF (95% CI)	Number of children with microhaematuria by Haemastix	Prevalence (%) of microhaematuria (95% CI)	Number of children tested for *S. mansoni*	Number of children positive with *S. mansoni*	Prevalence (%) of *S. mansoni* (95% CI)	Number of children tested for any species	Number of children egg-positive with any species	Prevalence (%) of infection with any species (95% CI)
Batie	478	53	11.1 (8.6–14.2)	47	9.8 (7.5–12.8)	480	1	0.2 (0.0–1.2)	478	53	11.1 (8.6–4.2)
Boromo	478	8	1.7 (0.9–3.3)	21	4.4 (2.9–6.6)	478	0	0 (0–0.8)	476	8	1.7 (0.9–3.3)
Boussé	479	6	1.3 (0.6–2.7)	16	3.3 (2.1–5.4)	480	0	0 (0–0.8)	479	6	1.3 (0.6–2.7)
Dano	480	13	2.7 (1.6–4.6)	23	4.8 (3.2–7.1)	480	0	0 (0–0.8)	480	13	2.7 (1.6–4.6)
Do	475	4	0.8 (0.3–2.1)	56	11.8 (9.2–15.0)	479	3	0.6 (0.2–1.8)	474	7	1.5 (0.7–3.0)
Garango	480	1	0.2 (0.0–1.2)	20	4.2 (2.7–6.3)	480	0	0 (0–0.8)	480	1	0.2 (0.0–1.2)
Karangasso-Vigué	478	12	2.5 (1.4–4.3)	63	13.2 (10.4–16.5)	480	0	0 (0–0.8)	478	12	2.5 (1.4–4.3)
Koudougou	478	0	0 (0–0.8)	4	0.8 (0.3–2.1)	476	0	0 (0–0.8)	474	0	0 (0–0.8)
Lena	480	0	0 (0–0.8)	49	10.2 (7.8–13.2)	480	1	0.2 (0.0–1.2)	480	1	0.2 (0.0–1.2)
Nanoro	480	3	0.6 (0.2–1.8)	11	2.3 (1.3–4.1)	479	0	0 (0–0.8)	479	3	0.6 (0.2–1.8)
Nongr-Massom	480	2	0.4 (0.1–1.5)	8	1.7 (0.8–3.3)	480	0	0 (0–0.8)	480	2	0.4 (0.1–1.5)
Pouytenga	480	25	5.2 (3.6–7.6)	22	4.6 (3.0–6.8)	480	0	0 (0–0.8)	480	25	5.2 (3.6–7.6)
Sig-Nonghin	478	14	2.9 (1.8–4.9)	29	6.1 (4.3–8.6)	478	0	0 (0–0.8)	476	14	2.9 (1.8–4.9)
Tenkodogo	480	28	5.8 (4.1–8.3)	33	6.9 (4.9–9.5)	480	0	0 (0–0.8)	480	28	5.8 (4.1–8.3)
Ziniaré	480	6	1.3 (0.6–2.7)	17	3.5 (2.2–5.6)	480	0	0 (0–0.8)	480	6	1.3 (0.6–2.7)
Zorgho	480	4	0.8 (0.3–2.1)	23	4.8 (3.2–7.1)	479	0	0 (0–0.8)	479	4	0.8 (0.3–2.1)
**Total**	**7664**	**179**	**2.3 (2.0**–**2.7)**	**442**	**5.8 (5.3**–**6.3)**	**7669**	**5**	**0.1 (0.0**–**0.2)**	**7653**	**183**	**2.4 (2.1**–**2.8)**

The overall prevalence of infection with any species ranged from 0% to 11.1% across the 16 HDs, where one HD (Batie) had a prevalence of >10%, nine HDs had a prevalence of between 1–10%, and six HDs had a prevalence of <1% (Table 1). Among 160 health areas surveyed, the prevalence of schistosomiasis of any species ranged from 0% to 62.5%, with 110 health areas having a prevalence of 0%, 40 health areas having a prevalence of 1–10%, and 7 health areas having a prevalence of >10% (S2 Table). The health areas of Batie and Midebdo in the Batie HD had a prevalence of 62.5%% and 45.8%, respectively.

**Fig 1:**
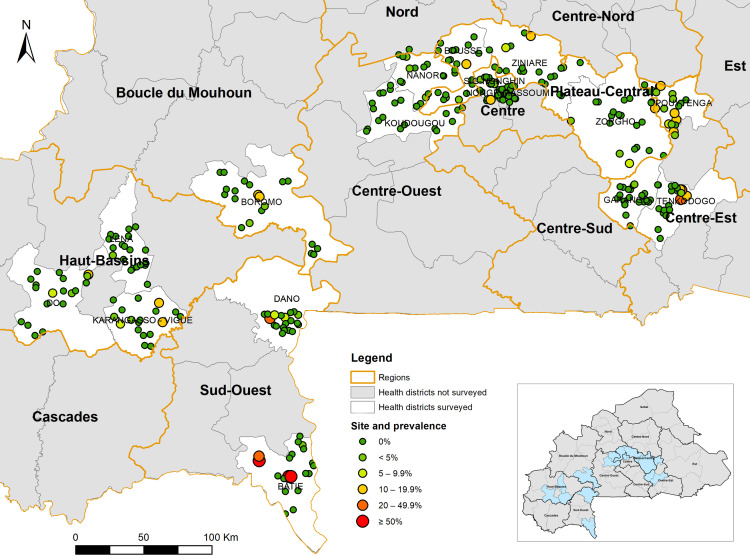
Geographical location of survey sites and point prevalence thresholds of schistosomiasis in 16 health districts in 2023–2024 in Burkina Faso. This map was created using the shapefiles of 70 health districts provided by the Ministry of Health of Burkina Faso (Ministry of Health and Public Hygiene, 2022, Burkina Faso Sanitary District 2022. Available at https://espen.afro.who.int/sites/default/files/content/cartography/files/ESPEN_IU_2022_0.zip) and the ArcGIS version 10.8.2 (ESRI, Redlands, California, United States).

**Fig 2:**
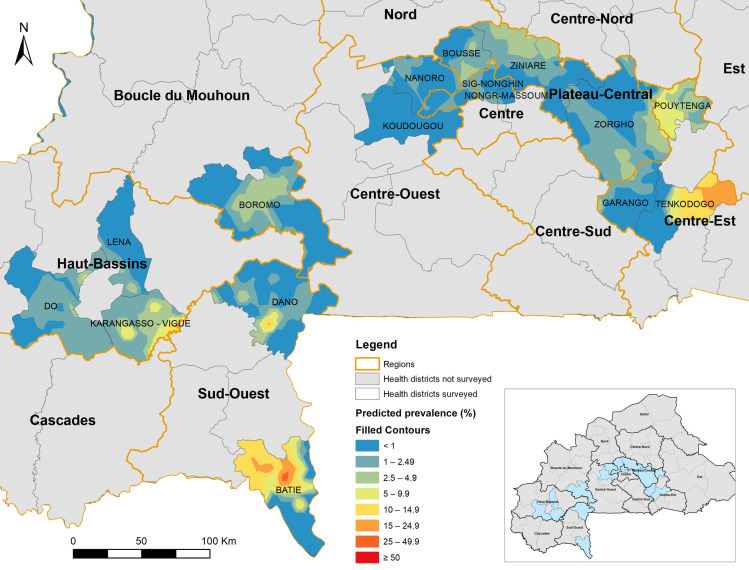
Smoothed contour map of predicted prevalence distribution of schistosomiasis in 16 health districts in 2023–2024 in Burkina Faso. This map was created using the shapefiles of 70 health districts provided by the Ministry of Health of Burkina Faso (Ministry of Health and Public Hygiene, 2022, Burkina Faso Sanitary District 2022. Available at https://espen.afro.who.int/sites/default/files/content/cartography/files/ESPEN_IU_2022_0.zip) and the ArcGIS version 10.8.2 (ESRI, Redlands, California, United States).

The overall prevalence of infection with any species ranged from 0% to 70.8% (median prevalence 0%) across the surveyed schools, with 256 schools having a prevalence of 0%, 40 schools 1–10%, and 24 schools >10% with three schools having a prevalence >50% (Fig 1). The schools with >10% prevalence are mostly located in districts (Pouytenga and Tenkodogo) in the East region and in the Batié district in the Sud Ouest region. Three schools with a prevalence of >50% are all located in the Batié district in the Sud Ouest region. The predicted prevalence distribution across the 16 HDs is shown in Fig 2. Several hotspots were identified in Batie, Tenkodogo, and Dano HDs with >95% confidence, while a few potential hotspots were located in Pouytenga, Bousse, and Karangasso-Vigué HDs with low (90%) confidence (figure not shown).

**Fig 3:**
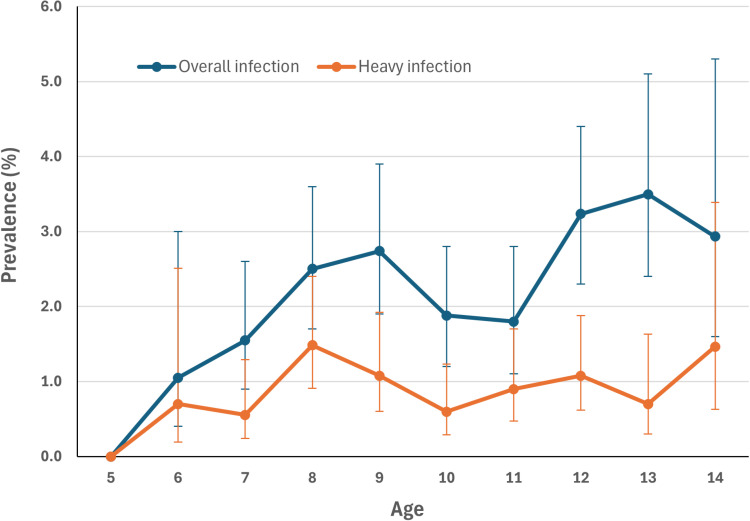
Overall prevalence and prevalence of HI infection by age across 16 health districts in 2023–2024 in Burkina Faso. Error bars represent 95% CI. 95% CI for prevalence in children aged five years was not shown as only 22 children of this age were tested with zero positive.

Overall, among age groups, the prevalence of infections with any species ranged from 0% (95% CI: 0–14.9%) in children aged 5 years (only 22 children tested) to 3.5% (95% CI: 2.4–5.1%) in children aged 13 years (Fig 3). There was no significant difference in prevalence by age (χ^2^ = 16.462, *df* = 9, *p* = 0.058). There were 3,815 boys and 3,838 girls examined. The prevalence in boys (3.1%, 95% CI: 6.0–7.6%) was slightly higher than that in girls (1.6%, 95% CI: 1.3–2.1%) (χ^2^ = 18.542, *p* < 0.001).

We extracted data from survey sites falling in any of these 16 HDs from previous surveys in 2004–2005, 2008–2013, and 2016–2019 (Table 2). As previous survey data were from different sites in these HDs and by different sampling methods, no statistical comparison could be made for differences in prevalence. Instead, we analyzed the trend of the prevalence in these HDs. We could not obtain a combined prevalence of schistosomiasis (*S. haematobium* and *S. mansoni*) from the previous surveys, and used the prevalence of *S. haematobium* infection as a proxy. The results showed a significant non-linear decline in the prevalence of *S. haematobium* infection during the four survey periods, using results from UF in 2023–2024 (Table 2), with an 89% reduction in 2023–2024 from 2004–2005.

**Table 2:** Average prevalence of *S. haematobium* infection in school-aged children from survey sites in any of the 16 districts in Burkina Faso.

Year of survey	Tests used	Number of children tested	Number of children positive with *S. haematobium* infection	Prevalence (%) of *S. haematobium* infection (95% CI)	Chi-square for linear trend	*P* value	Chi-square for non-linearity	*P* value
2004–2005	Urine filtration	1,922	391	20.3 (18.6–22.2)				
2008–2013	Urine filtration	4,012	323	8.1 (7.3–8.9)				
2016–2019	Urine filtration	2,553	32	1.3 (0.9–1.8)				
2023–2024	Urine filtration	7,664	179	2.3 (2.0–2.7)	728.105	0	340.427	0

### Intensity of infection

The mean intensity of infection for both species was low, 3.5 eggs/10ml (95% CI: 2.3–4.6 eggs/10ml) for *S. haematobium* and 0.3 epg (95% CI: 0–0.7 epg) for *S. mansoni* (Table 3). The prevalence of HI infection of any species was 0.9% (95% CI: 0.8–1.2%). Prevalence of HI infection ranged from 0% to 4.2% among 16 HDs, with four HDs (Tenkodogo, Batié, Sig-Nonghin, and Karangasso-Vigué) having a prevalence of HI infection over 1%. Tenkodogo had the heaviest infection with a prevalence of HI infection of 4.2% (95% CI: 2.7–6.4%). There were 29 of the 157 health areas surveyed had a prevalence of HI infection of >1%, ranging from 1.4% to 22.9%. Batie and Tenkodogo each had two health areas having a prevalence of HI infection of >10%: Batie (22.9%) and Midebdo (12.5%) in the Batie HD and Urban II (20.8%) and Urban I (12.5%) in the Tenkodogo HD. Among different ages, children aged 8, 9, 12, and 14 had a prevalence of HI infection of >1%. The prevalence of HI infection was significantly higher in boys (1.4%, 95% CI: 1.1–1.8%) than in girls (0.5%, 95% CI: 0.3–0.8%) (χ^2^ = 21.609, p < 0.001).

**Table 3:** Intensity of infection in 16 districts in 2023-2024 in Burkina Faso.

Districts surveyed	Number of children tested for *S. haematobium*	Number of children positive with *S. haematobium* by UF	Mean intensity of infection (eggs/10ml)	Number of children tested for *S. mansoni*	Number of children positive with *S. mansoni* by KK	Mean intensity of infection (epg)	Number of children tested for any species	Number of children with HI infection of any species	Prevalence (%) of HI infection with any species (95% CI)
Batie	478	53	11.0 (4.4–17.7)	480	1	1.7 (0–5.0)	478	18	3.8 (2.4–5.9)
Boromo	478	8	0.2 (0–0.5)	478	0	–	476	1	0.2 (0.0–1.2)
Boussé	479	6	0.4 (0–1.0)	480	0	–	479	2	0.4 (0.1–1.5)
Dano	480	13	4.8 (0–10.6)	480	0	–	480	4	0.8 (0.3–2.1)
Do	475	4	1.1 (0–3.1)	479	3	3.1 (0–9.0)	474	2	0.4 0.1–1.5)
Garango	480	1	0.0 (0–0.1)	480	0	–	480	0	0 (0–0.8)
Karangasso-Vigué	478	12	3.9 (0.6–7.2)	480	0	–	478	6	1.3 (0.6–2.7)
Koudougou	478	0	–	476	0	–	474	0	0 (0–0.8)
Lena	480	0	–	480	1	0.1 (0–0.4)	480	0	0 (0–0.8)
Nanoro	480	3	4.2 (0–10.0)	479	0	–	479	2	0.4 (0.1–1.5)
Nongr-Massom	480	2	0.7 (0–1.9)	480	0	–	480	2	0.4 (0.1–1.5)
Pouytenga	480	25	1.2 (0.4–2.0)	480	0	–	480	4	0.8 (0.3–2.1)
Sig-Nonghin	478	14	6.5 (1.0–12.1)	478	0	–	476	7	1.5 (0.7–3.0)
Tenkodogo	480	28	17.2 (5.8–28.7)	480	0	–	480	20	4.2 (2.7–6.4)
Ziniaré	480	6	3.9 (0–10.1)	480	0	–	480	3	0.6 (0.2–1.8)
Zorgho	480	4	0.4 (0–0.9)	479	0	–	479	1	0.2 (0.0–1.2)
**Total**	**7,664**	**179**	**3.5 (2.3**–**4.6)**	**7,669**	**5**	**0.3 (0**–**0.7)**	**7,653**	**72**	**0.9 (0.8**–**1.2)**

### Comparison of Haemastix and urine filtration results

**Fig 4:**
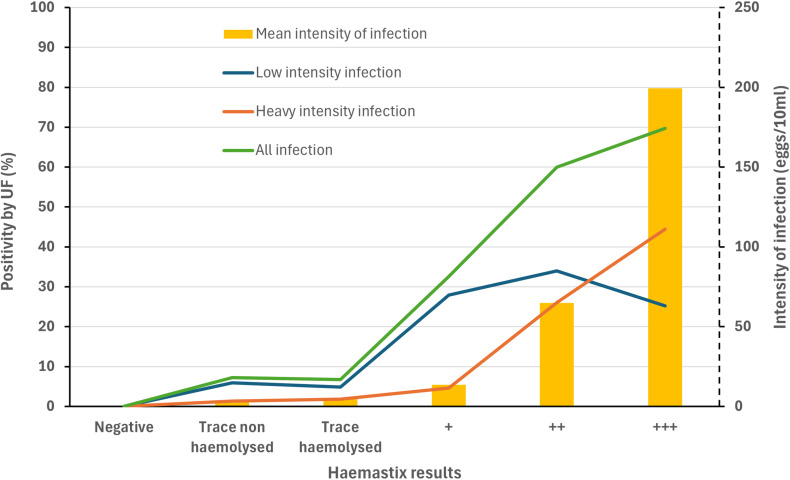
Relationship between Haemastix and urine filtration results in 16 health districts in 2023–2024 in Burkina Faso.

The prevalence of microhaematuria by Haemastix was 5.8% (5.3–6.3%), which was significantly higher than the prevalence of *S. haematobium* infection of 2.3% (2.0–2.7%) by UF (χ^2^ = 2112.70, p < 0.001). Haemastix results were significantly associated with the intensity of infection, where the results of the dipstick were stronger as the egg count increased (Pearson’s r = 0.361, p < 0.001) (Fig 4).

## Discussion

The impact assessment results in 2023–2024 showed that the overall prevalence of schistosomiasis was relatively low at 2.4% in the 16 HDs surveyed, with an overall prevalence of HI infection of 0.9%. There was a significant downward trend in the prevalence using *S. haematobium* infection as a proxy from 2004–2005 to 2023–2024 in these 16 HDs, a nearly 90% reduction from the baseline prevalence after two decades of good coverage of mass treatment. This suggests significant progress towards eliminating schistosomiasis as a public health problem in Burkina Faso. However, schistosomiasis continues to be a public health problem in some health areas in the surveyed districts, in particular, two health areas in Batie, two health areas in Tenkodogo, and one health area each in Boromo, Dano, and Sig-Nonghin had a prevalence of >10%. The highest site prevalence of 70,8% was in Batie health area in the Batie HD. These findings are supported by other studies carried out a decade after treatment that showed hotspots, where the prevalence was higher than 50% [11].

The prevalence of urogenital and intestinal schistosomiasis depended on the region. *S. haematobium* is found in almost all geographical areas of the country, while *S. mansoni* is only found in the Hauts-Bassins and Sud-Ouest regions. The distribution of schistosomiasis in the current surveys was in line with the previous surveys, with *S. haematobium* infection found in all 16 HDs and *S. mansoni* infection found in only three HDs (Batie, Do, and Lena). This could be explained by the fact that *S. haematobium* has several snail intermediate host species, whereas *S. mansoni* only has *Biomphalaria pfeifferii* as an intermediate host in Burkina Faso [9,24]. These results provided an updated geographical prevalence of schistosomiasis, with more granular prevalence estimates at the sub-district level in these districts for programmatic decisions.

The mean intensity of infection for both species was low, with an average prevalence of HI infection of 0.9% across 16 HDs. However, the HDs of Tenkodogo, Batié, Sig-Nonghin, and Karangasso-Vigué still had an average prevalence of HI infection over 1%, a threshold defined by WHO for reaching the elimination of schistosomiasis as a public health problem [3]. At the sub-district level, 29 health areas had a prevalence of HI infection of >1%, and particularly, two health areas in the Batie HD and two health areas in the Tenkodogo HD had a prevalence of HI infection of >10%. These districts need continued and enhanced efforts in treatment and, in particular, program actions, such as operational research to identify key risk factors and tailored treatment strategies, are needed in those health areas with a high prevalence of HI infection.

The Batié HD in the Sud Ouest region had the highest focal prevalence of 70.8% and had infections of both species. One survey site in 2004–2005 baseline mapping had a prevalence of *S. haematobium* infection of 55%, which was located in the Batie town, the same health area where two sites had the highest prevalence (70.8% and 62.5%) in the current survey, suggesting the prevalence of schistosomiasis has not been reduced but increased after eight rounds of MDA since 2004. Batié is an area with more surface water bodies, where intermediate snails can thrive. Rice growing is an agricultural activity in the area, which is one of the main sources of contamination of surface water [25,26]. The district also borders Côte d’Ivoire and Ghana, where schistosomiasis is also endemic [27,28]. Cross-border activities may have made treatment programs challenging. The national Neglected Tropical Disease Program has faced similar challenges in the same region for eliminating lymphatic filariasis and onchocerciasis. The previous assessments through sentinel sites across the country showed a great reduction in prevalence across Burkina Faso with the treatment strategy used [10,11]. However, Batie was not assessed in those surveys, and the situation in the district was unknown during those years. Communities with a high baseline prevalence of >50%, such as in Batie, need more frequent treatment, *i.e.*, as once a year in the WHO 2006 recommendations [14] and twice a year targeting whole communities as WHO currently recommends [3]. The treatment frequency every other year in Batie with irregular treatment of adults may not have been enough in this district.

Tenkodogo and Pouytenga HDs in the Centre Est region, Boromo HD in the Boucle du Mouhoun region, Sig-Nonghin HD in the Centre region, and Karangasso-Vigué HD in the Hauts Bassins region, where there were multiple survey sites with a prevalence of >10%, are located in areas with historically high prevalence of schistosomiasis [29]. Two sites with a high prevalence of HI infection in Tenkodogo HD are from the Tenkodogo urban area. The populations in these HDs concentrate around water reservoirs with a wide range of water contact activities, including farming, fishing, children’s bathing, washing clothes, and making building bricks. These activities are major risk factors for exposure to infections. Despite two decades of treatment, schistosome infections persist in some communities in these HDs. Treatment strategies need to be specifically tailored for communities with different levels of endemicity. Additional intervention strategies must be considered, such as focal snail management, health education for changing water contact behaviours, access to safe water and hygiene and sanitation facilities.

Urine filtration for detecting *S. haematobium* eggs and Haemastix for microhaematuria are both standard diagnostic methods to detect urogenital schistosomiasis [14,30,31]. The prevalence of microhaematuria by Haemastix was significantly higher than the prevalence of *S. haematobium* infection by urine filtration in our surveys. Studies showed similar results from others [32,33,34]. Haemastix results were closely related to the intensity of infection, as shown in Fig 4. The urine reagent strips remain a useful adjunct diagnostic test for rapid monitoring of urinary schistosomiasis in areas with low and high prevalence [17]. Other studies, however, have shown that in low-prevalence settings, microhaematuria is an unstable proxy for *S. haematobium* infections [35] because haematuria can be caused by a number of other conditions [36,37].

There are some limitations in this survey. First, we used a different sampling methodology in this survey from those used in previous surveys, and the survey sites were limited and in different locations in previous surveys. This made it inappropriate for direct statistical comparison. Therefore, the estimated prevalence reduction may not represent the actual situation in the 16 HDs. However, the previous assessment strategy using sentinel sites was insufficient and missed key information in communities such as in Batie. The current cluster sampling survey was more effective in providing valuable data to fill the data gaps and making programmatic decisions for sub-district level treatment. Second, the diagnostic tools used in these surveys all have low sensitivity in low-prevalence settings, treated populations, or groups with light-intensity infections [17,31,38]. These may have underestimated the true prevalence in these HDs. However, these are all valid diagnostic tools for program evaluation recommended by WHO [22]. These tools should effectively identify those HI infections and it is unlikely that the prevalence of HI infection reported in this paper was underestimated, which is a critical indicator for program evaluation.

## Conclusions

As a result of two decades of mass treatment, schistosomiasis may have been eliminated as a public health problem in 12 of the 16 HDs surveyed in Burkina Faso. However, some hotspots of infection remain in some areas of five HDs, with a prevalence of up to 70.8% (such as in Batie). The results enabled the national control program to shift to sub-district MDA in these HDs and focus efforts in persistent areas. The findings demonstrated the progress of schistosomiasis elimination in Burkina Faso but also revealed the importance of implementing tailored program strategies according to the local situation.

## Supporting information

S1 Table Programmatic coverage of praziquantel treatment in the 16 districts in Burkina Faso from 2004 to 2022. (DOCX)

S2 Table Prevalence of schistosomiasis by health areas in the 16 health districts in Burkina Faso in 2023-2024. (DOCX)

S1 Data Raw dataset used in this paper. (XLSX)

S1 Checklist STROBE checklist. (DOCX)
