# Supplementary material for: Epidemiological situation of schistosomiasis in 16 districts of Burkina Faso after two decades of mass treatment
Source: PLoS Negl Trop Dis. 2025 Feb 6;19(2):e0012858. doi: 10.1371/journal.pntd.0012858 (PMC11813138; doi:10.1371/journal.pntd.0012858)
Supplement: S1 Table — (DOCX) [file pntd.0012858.s001.docx]

**Table S1. Programmatic coverage of praziquantel treatment in the 16 districts in Burkina Faso from 2004 to 2022**

Note: Two rounds of MDA were conducted in the Centre Est region in 2015-2017 and the figures represent the coverage in the two rounds.
