# Supplementary material for: Epidemiological situation of schistosomiasis in 16 districts of Burkina Faso after two decades of mass treatment
Source: PLoS Negl Trop Dis. 2025 Feb 6;19(2):e0012858. doi: 10.1371/journal.pntd.0012858 (PMC11813138; doi:10.1371/journal.pntd.0012858)
Supplement: S2 Table — (DOCX) [file pntd.0012858.s002.docx]

**Table S2. Prevalence of schistosomiasis by health areas in the 16 health districts in Burkina Faso in 2023-2024**

| **District** | **Health area** | **Number of children tested** | **Number of children testing positive for any species** | **Prevalence (%) of infection with any species** | **Number of children with HI infection of any species** | **Prevalence (%) of HI infection of any species** |
| --- | --- | --- | --- | --- | --- | --- |
| Batié | Banaba | 48 | 0 | 0.0 | 0 | 0.0 |
| Batié | Batie | 48 | 30 | 62.5 | 11 | 22.9 |
| Batié | Bopiel | 48 | 0 | 0.0 | 0 | 0.0 |
| Batié | Dankana | 46 | 1 | 2.2 | 1 | 2.2 |
| Batié | Fadio | 48 | 0 | 0.0 | 0 | 0.0 |
| Batié | Koriba | 48 | 0 | 0.0 | 0 | 0.0 |
| Batié | Koudjo | 48 | 0 | 0.0 | 0 | 0.0 |
| Batié | Legmoin | 48 | 0 | 0.0 | 0 | 0.0 |
| Batié | Midebdo | 48 | 22 | 45.8 | 6 | 12.5 |
| Batié | Zindi | 48 | 0 | 0.0 | 0 | 0.0 |
| Boromo | Bana | 48 | 0 | 0.0 | 0 | 0.0 |
| Boromo | Kabourou | 48 | 0 | 0.0 | 0 | 0.0 |
| Boromo | Kahin | 48 | 0 | 0.0 | 0 | 0.0 |
| Boromo | Mamou | 48 | 0 | 0.0 | 0 | 0.0 |
| Boromo | Ouahabou | 47 | 1 | 2.1 | 0 | 0.0 |
| Boromo | Pa | 48 | 1 | 2.1 | 0 | 0.0 |
| Boromo | Pompoï | 48 | 0 | 0.0 | 0 | 0.0 |
| Boromo | Siby | 45 | 0 | 0.0 | 0 | 0.0 |
| Boromo | Tone | 48 | 0 | 0.0 | 0 | 0.0 |
| Boromo | Vy | 48 | 6 | 12.5 | 1 | 2.1 |
| Boussé | Bantogdo | 47 | 0 | 0.0 | 0 | 0.0 |
| Boussé | Koui | 48 | 0 | 0.0 | 0 | 0.0 |
| Boussé | Laye | 48 | 0 | 0.0 | 0 | 0.0 |
| Boussé | Napalgué | 48 | 0 | 0.0 | 0 | 0.0 |
| Boussé | Niou | 48 | 0 | 0.0 | 0 | 0.0 |
| Boussé | Sandogo srg | 48 | 1 | 2.1 | 1 | 2.1 |
| Boussé | Sao | 48 | 4 | 8.3 | 0 | 0.0 |
| Boussé | Sourgoubila | 48 | 1 | 2.1 | 1 | 2.1 |
| Boussé | Toeghin | 48 | 0 | 0.0 | 0 | 0.0 |
| Boussé | Urbain boussé | 48 | 0 | 0.0 | 0 | 0.0 |
| Dano | Bilbale | 48 | 10 | 20.8 | 4 | 8.3 |
| Dano | Dadone | 48 | 2 | 4.2 | 0 | 0.0 |
| Dano | Dissihn | 48 | 1 | 2.1 | 0 | 0.0 |
| Dano | Done | 48 | 0 | 0.0 | 0 | 0.0 |
| Dano | Kokolibou | 48 | 0 | 0.0 | 0 | 0.0 |
| Dano | Kpoperi | 48 | 0 | 0.0 | 0 | 0.0 |
| Dano | Mou | 48 | 0 | 0.0 | 0 | 0.0 |
| Dano | Nakar | 48 | 0 | 0.0 | 0 | 0.0 |
| Dano | Namare | 48 | 0 | 0.0 | 0 | 0.0 |
| Dano | Navrikpe | 48 | 0 | 0.0 | 0 | 0.0 |
| Do | Bouende | 48 | 0 | 0.0 | 0 | 0.0 |
| Do | Dogona | 48 | 0 | 0.0 | 0 | 0.0 |
| Do | Kiri | 47 | 4 | 8.5 | 1 | 2.1 |
| Do | Koumi | 48 | 2 | 4.2 | 1 | 2.1 |
| Do | Ksambla | 48 | 0 | 0.0 | 0 | 0.0 |
| Do | Logofoursso | 48 | 1 | 2.1 | 0 | 0.0 |
| Do | Nasso | 46 | 0 | 0.0 | 0 | 0.0 |
| Do | Peni | 46 | 0 | 0.0 | 0 | 0.0 |
| Do | Tapoko | 47 | 0 | 0.0 | 0 | 0.0 |
| Do | Toussiana | 48 | 0 | 0.0 | 0 | 0.0 |
| Garango | Boussouma | 48 | 0 | 0.0 | 0 | 0.0 |
| Garango | Dango | 48 | 0 | 0.0 | 0 | 0.0 |
| Garango | Fingla | 48 | 0 | 0.0 | 0 | 0.0 |
| Garango | Kombinatenga | 48 | 0 | 0.0 | 0 | 0.0 |
| Garango | Lengha | 48 | 0 | 0.0 | 0 | 0.0 |
| Garango | Niaogho 1 | 48 | 0 | 0.0 | 0 | 0.0 |
| Garango | Ouarégou | 48 | 0 | 0.0 | 0 | 0.0 |
| Garango | Sanogho | 48 | 0 | 0.0 | 0 | 0.0 |
| Garango | Toécé | 48 | 1 | 2.1 | 0 | 0.0 |
| Garango | Ziglakoulpélé | 48 | 0 | 0.0 | 0 | 0.0 |
| Karangasso-Vigué | Dan | 72 | 4 | 5.6 | 2 | 2.8 |
| Karangasso-Vigué | Deguelin | 48 | 0 | 0.0 | 0 | 0.0 |
| Karangasso-Vigué | Diosso | 48 | 0 | 0.0 | 0 | 0.0 |
| Karangasso-Vigué | Kvigue | 120 | 0 | 0.0 | 0 | 0.0 |
| Karangasso-Vigué | Poya | 48 | 3 | 6.3 | 2 | 4.2 |
| Karangasso-Vigué | Soumousso | 48 | 1 | 2.1 | 1 | 2.1 |
| Karangasso-Vigué | Wara | 48 | 3 | 6.3 | 1 | 2.1 |
| Karangasso-Vigué | Yeguere | 46 | 1 | 2.2 | 0 | 0.0 |
| Koudougou | Bingo | 47 | 0 | 0.0 | 0 | 0.0 |
| Koudougou | Boulsin | 47 | 0 | 0.0 | 0 | 0.0 |
| Koudougou | Cmk | 48 | 0 | 0.0 | 0 | 0.0 |
| Koudougou | Imasgo | 48 | 0 | 0.0 | 0 | 0.0 |
| Koudougou | Pehiri | 48 | 0 | 0.0 | 0 | 0.0 |
| Koudougou | Pitmoaga | 47 | 0 | 0.0 | 0 | 0.0 |
| Koudougou | Ramongo | 47 | 0 | 0.0 | 0 | 0.0 |
| Koudougou | Rana | 47 | 0 | 0.0 | 0 | 0.0 |
| Koudougou | Sakoinse | 47 | 0 | 0.0 | 0 | 0.0 |
| Koudougou | Some | 48 | 0 | 0.0 | 0 | 0.0 |
| Lena | Bah | 48 | 0 | 0.0 | 0 | 0.0 |
| Lena | Bala | 48 | 0 | 0.0 | 0 | 0.0 |
| Lena | Dorossiamasso | 24 | 0 | 0.0 | 0 | 0.0 |
| Lena | Kadomba | 72 | 0 | 0.0 | 0 | 0.0 |
| Lena | Kofila | 48 | 0 | 0.0 | 0 | 0.0 |
| Lena | Koroma | 24 | 0 | 0.0 | 0 | 0.0 |
| Lena | Lena | 96 | 0 | 0.0 | 0 | 0.0 |
| Lena | Sala | 48 | 1 | 2.1 | 0 | 0.0 |
| Lena | Satiri | 48 | 0 | 0.0 | 0 | 0.0 |
| Lena | Tiarako | 24 | 0 | 0.0 | 0 | 0.0 |
| Nanoro | Cm urbain | 72 | 1 | 1.4 | 1 | 1.4 |
| Nanoro | Godo | 24 | 0 | 0.0 | 0 | 0.0 |
| Nanoro | Kindi | 47 | 1 | 2.1 | 0 | 0.0 |
| Nanoro | Kone | 48 | 0 | 0.0 | 0 | 0.0 |
| Nanoro | Kouria | 48 | 0 | 0.0 | 0 | 0.0 |
| Nanoro | Lalle | 48 | 0 | 0.0 | 0 | 0.0 |
| Nanoro | Nassoulou | 48 | 0 | 0.0 | 0 | 0.0 |
| Nanoro | Nazoanga | 48 | 1 | 2.1 | 1 | 2.1 |
| Nanoro | Soaw | 48 | 0 | 0.0 | 0 | 0.0 |
| Nanoro | Somassi | 48 | 0 | 0.0 | 0 | 0.0 |
| Nongr-Massom | Banporé | 48 | 0 | 0.0 | 0 | 0.0 |
| Nongr-Massom | Polesgo | 48 | 1 | 2.1 | 1 | 2.1 |
| Nongr-Massom | Roumtenga | 72 | 0 | 0.0 | 0 | 0.0 |
| Nongr-Massom | Sakoula | 48 | 0 | 0.0 | 0 | 0.0 |
| Nongr-Massom | Secteur 13 | 48 | 0 | 0.0 | 0 | 0.0 |
| Nongr-Massom | Secteur 23 | 48 | 0 | 0.0 | 0 | 0.0 |
| Nongr-Massom | Secteur 25 | 48 | 0 | 0.0 | 0 | 0.0 |
| Nongr-Massom | Secteur 26 | 48 | 1 | 2.1 | 1 | 2.1 |
| Nongr-Massom | Secteur 27 | 48 | 0 | 0.0 | 0 | 0.0 |
| Nongr-Massom | Sogdin | 24 | 0 | 0.0 | 0 | 0.0 |
| Pouytenga | Balkiou | 24 | 2 | 8.3 | 0 | 0.0 |
| Pouytenga | Belme | 48 | 4 | 8.3 | 1 | 2.1 |
| Pouytenga | Doundoudougou | 48 | 0 | 0.0 | 0 | 0.0 |
| Pouytenga | Kando | 48 | 4 | 8.3 | 0 | 0.0 |
| Pouytenga | Kodemende | 24 | 0 | 0.0 | 0 | 0.0 |
| Pouytenga | Mobega | 72 | 1 | 1.4 | 0 | 0.0 |
| Pouytenga | Naryaoghin | 72 | 5 | 6.9 | 2 | 2.8 |
| Pouytenga | Pissy | 48 | 4 | 8.3 | 1 | 2.1 |
| Pouytenga | Tambogo | 48 | 3 | 6.3 | 0 | 0.0 |
| Pouytenga | Tanga | 48 | 2 | 4.2 | 0 | 0.0 |
| Sig-Nonghin | Bassinko | 48 | 0 | 0.0 | 0 | 0.0 |
| Sig-Nonghin | Bissighin | 46 | 6 | 13.0 | 3 | 6.5 |
| Sig-Nonghin | Cooperation allemande | 48 | 0 | 0.0 | 0 | 0.0 |
| Sig-Nonghin | Bilgo | 24 | 1 | 4.2 | 0 | 0.0 |
| Sig-Nonghin | Goupana | 24 | 0 | 0.0 | 0 | 0.0 |
| Sig-Nonghin | Nedogo | 24 | 0 | 0.0 | 0 | 0.0 |
| Sig-Nonghin | Sabtenga | 47 | 0 | 0.0 | 0 | 0.0 |
| Sig-Nonghin | Saint joseph | 119 | 7 | 5.9 | 4 | 3.4 |
| Sig-Nonghin | Kamboincin | 48 | 0 | 0.0 | 0 | 0.0 |
| Sig-Nonghin | Zibako | 48 | 0 | 0.0 | 0 | 0.0 |
| Tenkodogo | Bagre perimetre | 48 | 0 | 0.0 | 0 | 0.0 |
| Tenkodogo | Cmu | 48 | 2 | 4.2 | 1 | 2.1 |
| Tenkodogo | Loanga cella | 48 | 0 | 0.0 | 0 | 0.0 |
| Tenkodogo | Oueguedo | 48 | 0 | 0.0 | 0 | 0.0 |
| Tenkodogo | Ounzeogo | 48 | 0 | 0.0 | 0 | 0.0 |
| Tenkodogo | Sabtenga | 48 | 0 | 0.0 | 0 | 0.0 |
| Tenkodogo | Urbain i | 48 | 8 | 16.7 | 6 | 12.5 |
| Tenkodogo | Urbain ii | 48 | 14 | 29.2 | 10 | 20.8 |
| Tenkodogo | Urbain iii | 48 | 4 | 8.3 | 3 | 6.3 |
| Tenkodogo | Zabo | 48 | 0 | 0.0 | 0 | 0.0 |
| Ziniaré | Dispensaire moutti | 48 | 0 | 0.0 | 0 | 0.0 |
| Ziniaré | Donsin | 48 | 0 | 0.0 | 0 | 0.0 |
| Ziniaré | Kolokom | 48 | 0 | 0.0 | 0 | 0.0 |
| Ziniaré | Laongo | 48 | 0 | 0.0 | 0 | 0.0 |
| Ziniaré | Nioniogo | 48 | 0 | 0.0 | 0 | 0.0 |
| Ziniaré | Ourgou | 48 | 2 | 4.2 | 1 | 2.1 |
| Ziniaré | Sadaba | 48 | 3 | 6.3 | 2 | 4.2 |
| Ziniaré | Sawana | 48 | 1 | 2.1 | 0 | 0.0 |
| Ziniaré | Tampaongo | 48 | 0 | 0.0 | 0 | 0.0 |
| Ziniaré | Urbain | 48 | 0 | 0.0 | 0 | 0.0 |
| Zorgho | Bourma | 48 | 3 | 6.3 | 1 | 2.1 |
| Zorgho | Dawaka | 48 | 0 | 0.0 | 0 | 0.0 |
| Zorgho | Koumséogo | 48 | 0 | 0.0 | 0 | 0.0 |
| Zorgho | Meguet | 48 | 0 | 0.0 | 0 | 0.0 |
| Zorgho | Mogtedo | 72 | 1 | 1.4 | 0 | 0.0 |
| Zorgho | Sankuissi | 48 | 0 | 0.0 | 0 | 0.0 |
| Zorgho | Talembika | 72 | 0 | 0.0 | 0 | 0.0 |
| Zorgho | Wada | 47 | 0 | 0.0 | 0 | 0.0 |
| Zorgho | Zorgho | 48 | 0 | 0.0 | 0 | 0.0 |
